# Socioeconomic inequality in timing of ANC visit among pregnant women in Ethiopia, 2019

**DOI:** 10.3389/fpubh.2024.1243433

**Published:** 2024-03-14

**Authors:** Atitegeb Abera Kidie, Desale Bihonegn Asmamaw, Tadele Biresaw Belachew, Samrawit Mihret Fetene, Tsegaw Amare Baykeda, Abel Endawkie, Alebachew Ferede Zegeye, Tadesse Tarik Tamir, Sisay Maru Wubante, Elsa Awoke Fentie, Wubshet Debebe Negash, Banchilay Addis

**Affiliations:** ^1^Department of Public Health, College of Health Science, Woldia University, Woldia, Ethiopia; ^2^Department of Reproductive Health, Institute of Public Health, College of Medicine and Health Sciences, University of Gondar, Gondar, Ethiopia; ^3^Department of Health Systems and Policy, Institute of Public Health, College of Medicine and Health Sciences, University of Gondar, Gondar, Ethiopia; ^4^Department of Epidemiology and Biostatistics, School of Public Health, College of Medicine and Health Science, Wollo University, Dessie, Ethiopia; ^5^Department of Medical Nursing, School of Nursing, College of Medicine and Health Sciences, University of Gondar, Gondar, Ethiopia; ^6^Department of Pediatric and Child Health Nursing, School of Nursing, College of Medicine and Health Sciences, University of Gondar, Gondar, Ethiopia; ^7^Department of Health Informatics, Institute of Public Health, College of Medicine and Health Sciences, University of Gondar, Gondar, Ethiopia

**Keywords:** antenatal care, timing, inequality, pregnant women, Ethiopia

## Abstract

**Background:**

Antenatal care (ANC) remains an invaluable approach to preventive care for ensuring maternal and infant health outcomes. Women in sub-Saharan Africa tend to delay their first antenatal care visits. In Ethiopia, only 20% of women received their first antenatal care during the first trimester of pregnancy. Timely and appropriate antenatal care practices can potentially save the lives of both mothers and children. Understanding socioeconomic inequality in the timing of antenatal care visits and its determinants may contribute to tackling disparities and achieving the sustainable development goals for maternal health.

**Objective:**

This study aimed to assess the socioeconomic inequality in the timing of antenatal care visit.

**Method:**

Secondary data sourced from the Mini Ethiopian Demographic Health Survey 2019 were used for this study. A total of 2,906 pregnant women were included in the study, and concentration curves were used to show inequality among sociodemographic and economic variables. Decomposition analysis was performed to estimate the contribution of each independent variable to the inequality in the timing of antenatal care visits.

**Result:**

The estimate of early initiation of antenatal care was 63%. The concentration index was 0.18 (*P* < 0.001). The inequality in the timing of antenatal care visit was more concentrated among the wealthiest pregnant women with a concentration index value of 0.18 (*P* < 0.001). Based on decomposition analysis results, the wealth index (81.9%.), education status (22.29%), and region (0.0642%) were identified as contributing factors to the inequality in the timing of antenatal care visits among women.

**Conclusion:**

The wealth index, educational status, and region were significant contributors to inequality in the early initiation of antenatal care visit. Improving women's wealth and education and narrowing the inequality gap are crucial for improving the health status of women and their children. We should focus on interventions targeted at early antenatal care visit to address the determinants of socioeconomic inequities.

## Introduction

Globally, pregnancy-related complications accounts for 300,000 maternal deaths (1). Maternal mortality is a major health problem in sub-Saharan Africa, and Ethiopia is one of the countries with the highest maternal mortality ratio of 412/100,000 (2, 3).

Antenatal care (ANC) represents a healthcare service provided to pregnant women for early prevention, diagnosis, and treatment of medical and pregnancy-related complications (4). Globally, ANC remains an extremely useful approach to preventing maternal and infant health outcomes (5). Improved and high-quality ANC is important for pregnant women and is crucial for the health of mother and child during pregnancy (6). The global prevalence of early initiation of ANC visits was 43% (3). Even though the World Health Organization (WHO) recommends that pregnant women in developing countries should start their visits before or at 16 weeks of gestation, the prevalence of late ANC visit is high in sub-Saharan Africa, and its magnitude ranges from 53% to 89% (2, 7, 8). In Ethiopia, focused ANC is provided freely; however, only a limited percentage (20%) of women had their first ANC visits early (3, 6). According to the EDHS 2011 report, only 11% of pregnant women received their initial ANC service within the recommended timeframe during their pregnancy (2).

The life-saving strategy for both mothers and children is a timely antenatal care visit (1). This is mainly attributed to the availability of various services and interventions available to specific stages of pregnancy (9). Early ANC visit enables women to meet WHO recommendations and is one of the major requirements of WHO's positive pregnancy experience (7). Early initiation of ANC visit helps health professionals provide timely information about health conditions for pregnant women and give health services according to their gestational age. In contrast, mothers who did not attend early ANC lose the opportunity to receive vital health information and interventions (10). Early initiation of the ANC is one valuable method in the early detection of undesirable pregnancy outcomes, such as low birth weight, stillbirth, intrauterine fetal death, and other complications (6). The timing of ANC visits is crucial for reducing maternal and child deaths (11). ANC offers the chance to provide preventive health services such as tetanus immunization, malaria, and worm treatment, as well as HIV testing and counseling, which can prevent mother-to-child HIV transmission. To maximize these benefits, early initiation of ANC is crucial for pregnant women (12). Previous research showed that unintended pregnancy, maternal knowledge, educational status of women, household socioeconomic status, maternal age, level of education, husband occupation, low monthly income, residence, and parity were determinants of timely booking of antenatal care (13–15).

Despite increased ANC visits and the quality of services received in Ethiopia, many women still need to start their first ANC visit early (6). Understanding socioeconomic inequality in the timing of ANC visits and its predictors may contribute to tackling disparities and achieving the SDGs for maternal health. Studies conducted in Ethiopia were regarding the actual determinants of early ANC visit and the inequality gaps in terms of socioeconomic factors were not studied. Moreover, in Ethiopia, little is known about socioeconomic inequality in the timing of ANC visit. Therefore, this study was aimed at identifying socioeconomic inequalities in the timing of the first ANC visit among Ethiopian women.

## Methodology

### Study area and period

This study used secondary data from the Ethiopia Mini Demographic and Health Survey (EMDHS) sourced online from a website: https://www.dhsprogram.com. The EMDHS 2019 is a cross-sectional study and it is the second Mini Demographic and Health Survey conducted in Ethiopia. Administratively, Ethiopia is divided into nine geographical regions and two administrative cities. This study was conducted from 21 March 2019 to 28 June 2019.

### Sampling technique and procedure

This study was conducted based on the secondary data from the Ethiopia Mini Demographic and Health Survey 2019. The EMDHS was conducted using a two-stage cluster sampling technique. Each region of the country was grouped into urban and rural strata. In the first stage of sampling, a total of 305 enumeration areas (EAs) were selected using the probability proportional allocation method by considering the enumeration area size. A household listing operation was carried out in all selected EAs from January 2019 through April 2019. The resulting lists of households served as a sampling frame for the selection of households in the second stage. Some of the selected EAs for the 2019 EMDHS were large, with more than 300 households. To minimize the task of household listing, each large EA selected for the 2019 EMDHS was segmented. Only one segment was selected for the survey, with probability proportional to segment size. Household listing was conducted only in the selected segment, that is, a 2019 EMDHS cluster is either an EA or a segment of an EA.

In the second stage, a fixed number of 30 households per cluster was selected by equally allocating for clusters and systematically selecting households from the newly created household listing. All women between the ages of 15–49 years old, either permanent residents of the selected households or visitors who slept in the household the night before the survey, were included in this study (16).

### Study variables

The variable of interest was socioeconomic inequality in the timing of ANC visit. The timing of the ANC visit was categorized into early and late visit. An early ANC visit is operationalized as attending the first ANC at the 4^th^ month of pregnancy or earlier, while a late visit is when a woman schedules ANC after 4 months of pregnancy. The outcome variable was dichotomized and coded as “1” for early visits and “0” for late visits. The independent variables of the study were age, residence, wealth index, region, education level, and marital status of women. For categorizing wealth index, households were given scores based on the quantity and types of consumer goods they own, ranging from a television to a bicycle or car, as well as their housing characteristics such as the source of drinking water, toilet facilities, and flooring materials. These scores are derived using principal component analysis. National wealth quintiles were compiled by assigning the household scores to each usual household member, ranking each person in the household population based on their scores and then dividing the distribution into five equal categories (richest, richer, middle, poorer, and poorest) (16).

### Data management and analysis

The extracted data were cleaned and categorized, and weighting of the data was performed using the women sample weight. In order to ensure a representative sample of Ethiopia, the distribution of the women in the sample was weighted. Some regions were overrepresented and some regions were underrepresented. The regions with small population were oversampled. To adjust for this sampling, we used weighting of samples with women sample weight divided by 1,000,000. Sample weights are calculated to six decimals but are presented in the standard recode files without the decimal point. It must be divided by 1,000,000 before use to approximate the number of cases (17).

The missing data were identified and were deleted or excluded. Finally, the cleaned data were analyzed using Stata version 14. Depending on the type of variables, descriptive statistics were carried out and reported using frequency, percentages, mean, and standard deviation. The results of this study were reported using text, tables, and figures.

In this study, concentration curves (CC) were used to assess whether there is socioeconomic inequality in the timing of ANC or not. CC represent the cumulative percentage of the dependent variable against the cumulative percentage of the population ranked by the socioeconomic status, typically the wealth index. A straight diagonal line serves as a reference for equality. If the concentration curve is above this diagonal line, it means the outcome variable is more concentrated among poorer individuals. Conversely, if it lies below the diagonal line, it indicates a concentration among wealthier individuals (18). However, a concentration curve is not enough to measure the magnitude of inequality. To measure the magnitude of socioeconomic inequality in the timing of ANC visit of pregnant women in Ethiopia, the Erreygers concentration index (CI) was used. The concentration index (CI) ranges from −1 to 1. A CI value of zero signifies no inequality in the distribution of the outcome by wealth, aligning with the line of inequality. A negative CI indicates disproportionate concentration of the outcome among the poorest, while a positive CI suggests inequality concentrated among the richest. The closer the absolute value of the CI is to one, the higher the level of inequality (19). The final analysis was performed using decomposition analysis to identify the contribution of each socioeconomic variable to the inequality of timing of ANC visits. Decomposition analysis was used to assess socioeconomic inequality because it provides methods for understanding the underlying factors contributing to socioeconomic inequalities in the timing of ANC visit and quantifying the respective contribution of factors. The variables with a *p*-value of < 0.05 were considered significant contributors to the inequality of the outcome variable.

## Results

### Descriptive statistics

This study included 2,906 pregnant women. Using this larger sample size increases the statistical power and improves the precision of effect size estimates. The study is more likely to detect small effect sizes, leading to greater generalizability to the broader population of Ethiopian pregnant women.

Out of the total number of pregnant women, 2,011 (69.2%) of them were from rural areas. Nearly half of the respondents, 1,200 (41.3%), were Muslim, and 1,083 (37.3%) of them were orthodox followers. The average age of women was 28 years with standard deviation of (SD ± 9). The majority of the women, 1,541 (53%), were within the age group of 25–34 years. Regarding the region, nearly half of the respondents (1,319) 45.4%, were from large central regions. Regarding the education level of participants, approximately 1,255 (43.2%) of the women had secondary and above education levels, and almost all of them, 2,662 (91.6%), were married (Table 1).

**Table 1:** Sociodemographic characteristics of pregnant women in Ethiopia, 2019 (*N* = 2,906).

**Variables**	**Category**	**Unweighted frequency**	**Weighted frequency**	**Unweighted percent**
Residence	Urban	895	866	30.8
	Rural	2,011	2,028	69.2
Religion	Orthodox	1,083	1,204	37.3
	Protestant	581	785	19.9
	Muslim	1,200	874	41.3
	Others	42	31	1.5
Age category	15–19	170	153	5.8
	20–24	607	596	21
	25–34	1,541	1,538	53
	35–49	588	607	20.2
Region	Metropolitan	706	149	24.3
	Large central	1,319	2,592	45.4
	Small peripheral	881	153	30.3
Wealth index	Poor	1,087	973	37.4
	Middle	455	585	15.7
	Rich	1,364	1,336	46.9
Number of ANC	< 4	2,803	2,775	96.5
	≥4	103	119	3.5
Education status	No education	585	1,264	20.1
	Primary	1,066	1,148	36.7
	Secondary and above	1,255	482	43.2
Marital status	Married	2,662	2,706	91.6
	Other	244	187	8.4
Number of living children	No	32	27	1.1
	1–3	1,794	1,763	61.7
	4 and above	1,080	1,104	37.2

### Timing of ANC visit by sociodemographic characteristics of participants (weighted *N* = 2,894)

Regarding the education level, 675 (23.3%) pregnant women with no formal education, 763 (26.4%) women with primary education, and 386 (13.3%) women with secondary and above education had timely ANC visits. Regarding the region, 91 (3.1%) pregnant women from small peripheral regions, 125 (4.3%) women from metropolitan regions and 1,608 (55.6%) from large central regions had timely ANC visit. Approximately 502 (17.3%) women with poor wealth index, 371 (12.9%) with middle wealth index, and 951 (32.8%) with rich wealth index had timely ANC visit.

### Timing of ANC visit

Approximately 63% (95% CI: 0.61, 0.65) of the pregnant women attended their ANC visit timely (4 months and before). In terms of their pregnancy trimesters, 1,824 (63%) pregnant women attended during the first trimester, 988 (34%) of them attended during the second trimester, and the remaining 81 (3%) of them during the third trimester.

### Socioeconomic inequality in timing of ANC visit

**Figure 1:**
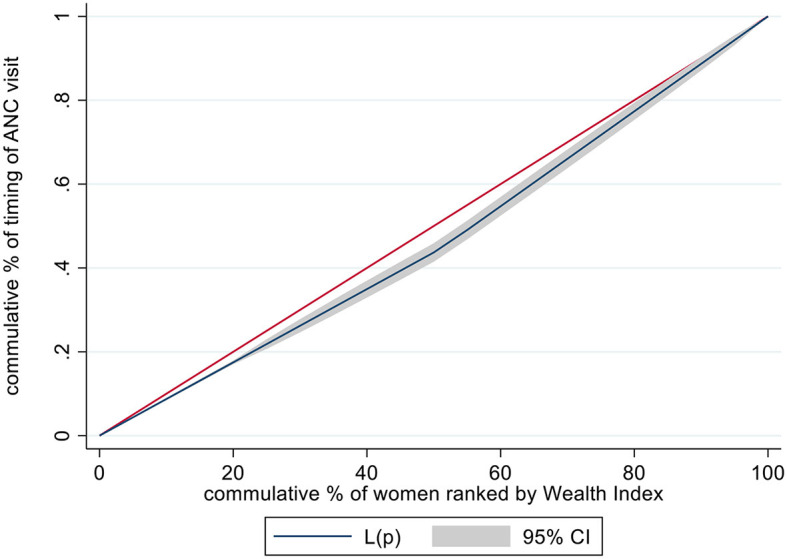
Concentration curve illustrates the inequality in the timing of ANC visit among pregnant women in Ethiopia, 2019, based on their wealth status.

The socioeconomic inequalities were assessed using a concentration curve, and this finding showed that the curve lay below the diagonal linear line. This explained that early ANC visit was more concentrated among the wealthiest women. The concentration curve finding is supported by a concentration index value of 0.18 (*P* < 0.001) (Figure 1).

**Figure 2:**
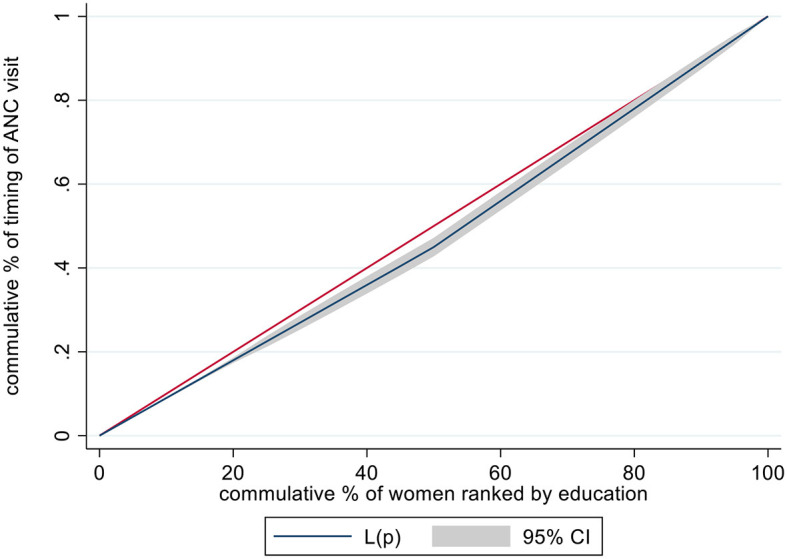
Concentration curve illustrates the inequality in the timing of ANC visit among pregnant women in Ethiopia, 2019, based on their education level.

The concentration curve showed that there were inequalities in the timing of ANC visits with respect to educational levels. More concentration was seen among pregnant women with higher education status. The *p*-value of < 0.001 with an index value of 0.2 supports the inequalities and is in favor of educated women (with secondary and above education levels) (Figure 2).

### Decomposition analysis on socioeconomic inequality in the timing of ANC visit

In the decomposition analysis results, coefficients, elasticity, concentration index, absolute contribution, and percentage contribution of each socioeconomic and demographic variable were estimated. The variables such as region, education level, and wealth index were significant contributors to inequality in the timing of ANC visit. This finding showed that the wealth index was the major contributor to inequality in the timing of ANC visit, which accounted for 81.9%. The second variable was the education level, contributing to 22.29% of inequality in the timing of ANC visit, specifically 3.48% from primary education levels and 18.81% from secondary and above education levels. The third significant variable was region, which contributed to 0.0642% of inequality in the timing of ANC visit. Generally, these three factors result in increasing the inequality in the timing of ANC visit.

This study finding showed that the region and inequality in the timing of ANC visit had significant relationship. The concentration index shows that the inequality in the timing of ANC visits among pregnant women from metropolitan regions was more concentrated on the rich households (CI = 0.1193). However, among small peripheral regions, the inequality in early ANC visit was concentrated more on the poor households (CI = −0.0869). The socioeconomic inequality of primary (CI = 0.0428), secondary and above education (CI = 0.2622) levels was more concentrated on the wealthiest households.

The elasticity of regions showed that a 1% change in large central to metropolitan regions resulted in a 3.1% increment in inequality in the timing of ANC visit, contributing to only 0.004% of socioeconomic inequality. While a 1% change in large central to small peripheral regions resulted in a 0.0013% decrement in inequality in the timing of ANC visit. Similarly, a 1% change in no formal education to primary, secondary and above education levels resulted in a 0.1484 (14.84%) and 13.1% increment in socioeconomic inequality in the timing of ANC visit, respectively. Although residence was not a significant contributor, a 1% change in urban to rural residence results in a 20.7% decrement in inequality in the timing or early ANC visit (Table 2).

**Table 2:** Factors contributing to the inequality in timing of ANC visit among pregnant women in Ethiopia, 2019.

**List of variables**	**Categories**	**Coefficient**	**Elasticity**	**Concentration index**	**Absolute contribution**	**Percentage contribution**
Residence	Urban (ref)	-	-	-	-	-
	Rural	−0.0765	−0.2074	−0.5060	0.1049	57.59
Region	Large central (ref)	-	-	-	-	-
	Metropolitan	0.1286^*^	0.0309	0.1193	0.0037	0.004
	Small peripheral	−0.0043^*^	−0.0013	−0.0869	0.0001	0.0602
Subtotal					0.0038	0.0642
Marital status	Not married (ref)	-	-	-	-	-
	Married	0.0688	0.1776	−0.1263	−0.0224	−12.306
Age	15–19 (ref)	-	-	-	-	-
	20–24	0.0008	0.0005	0.0456	0.00002	0.0132
	25–34	0.0109	0.0139	−0.0235	−0.0003	−0.1795
	35–49	−0.0337	−0.0359	−0.0272	0.001	0.537
Sub total					0.00072	0.3707
Education level	No formal education (ref)	-	-	-	-	-
	Primary	0.0889^*^	0.1484	0.0428	0.006	3.48
	Secondary and above	0.1821^*^8	0.1308	0.2622	0.0344	18.81
Sub total					0.0404	22.29
Wealth index	Poor(ref)	-	-	-	-	-
	Middle	0.0937^*^	0.0706	−0.0945	**−0**.007	−3.66
	Rich	0.0836^*^	0.1565	0.996	0.156	85.56
Subtotal					0.149	81.9

^*^p-value < 0.05, ref, reference.

## Discussion

This study investigates the socioeconomic inequality in the timing of ANC visit and its determinants among pregnant women in Ethiopia, which is essential for appropriate interventions directed to reduce maternal mortality and to achieve sustainable development goal (SDG 3). This study finding showed that approximately 63% of women had scheduled their ANC visits timely (4 months and earlier). This study finding was higher than those of studies carried out in Bahir Dar city (44.2%), Sodo(39%), west Gojam (31.5%), Ethiopia (26.8%), Nigeria (24%), southeastern Tanzania (29%), Malawi (28%), Gondar (47.4%), and Tigray region (27.5%) (1, 3–5, 7, 13, 20–22). Similarly, these findings were higher than the results of the study conducted based on the 2016 EDHS that approximately 20% of the mothers initiated ANC timely, Bangladesh (43%) and South Sudan (14.1%) (6, 23, 24). However, this was lower than the study finding of Nepal that showed 70% of women had started their first ANC at 4 months or earlier (10). This difference in the magnitude of the timing of ANC visits may be attributed to the difference in the study setting, socioeconomic and demographic status, sociocultural factors, and sample size.

In the decomposition analysis, the region, educational status, and wealth index were significant factors contributing to inequality in the timing of ANC visit. According to this study results, the inequalities in the timing of ANC visit inclined to the wealthier, more educated pregnant women from small peripheral and metropolitan regions.

This study finding showed that the wealth index was the major contributor to the timing of ANC visit. The inequality gap by wealth index results in a significant reduction in timing of ANC visit. Based on this study's findings, inequality in the timing of ANC visit was in favor of women in poor households. This was in line with the study conducted in Tanzania, Malawi, Cameroon, Ghana Nepal, Ethiopia, Oromia region, Debre Birhan, SADC countries, and Oromia based on 2016 EDHS and developing countries. These studies showed that the wealth index was the predominant contributing factor to economic-related inequality gaps in the timing of ANC visit (10, 11, 21, 25–31). Furthermore, this study's finding were consistent with those conducted in countries of South Asia and sub-Saharan Africa, which showed that mothers with poor economic status were significant predictors of inequality in ANC visits (30).

Mothers from low-income households could skip ANC appointments because they can't afford dependable public or private transportation. Due to this, there may be delay in receiving ANC services. Therefore, reducing financial barriers to timely use of ANC services reduces the inequality gap among women. The other possible reason and the indirect effect might be the case that, most of the time, wealthier women have higher levels of education and greater access to information about ANC and its importance. They are more likely to understand the benefits of ANC visits and finally they can decide to seek ANC services early in their pregnancies. Therefore, addressing the gaps in the timing of ANC visit in terms of the wealth index needs efforts to make all socioeconomic groups initiate their ANC early and equally.

The educational status of women was another important and significant contributor to socioeconomic inequality in early initiation of ANC, which accounts for 22.29% of the contributing factors. Women who had primary, secondary, and above education levels showed an increment in the timing of ANC visits, contributing to the inequality gap. This finding is consistent with the study conducted in Ethiopia, which showed that the maternal educational status was the second dominant driver, accounting for 28.48% of the contributing factors (26). This is in line with the study conducted in Tanzania, Debre Birhan, Gondar, and countries of South Asia and sub-Saharan Africa (22, 25, 28, 30, 32).

This study finding is also consistent with a study conducted in the Oromia region based on the 2016 EDHS, as well as studies carried out in Vietnam and Malawi, which revealed that the educational status of women is a significant contributing factor to inequality (21, 33). Similarly, the findings of the study conducted in Nepal indicated that women with less educated backgrounds tend to contribute to inequalities in timely ANC initiation (34).

This is because educated pregnant women might have a better socioeconomic status that helps them have financial autonomy to visit ANC and have a better access to information, which allow women to make informed and positive decisions to visit at the recommended time (26). Women with higher education may be more likely to live in urban areas, gain employment and wealth, and have a better understanding of the benefits of receiving ANC services.

The other reason for women having access to ANC could be their educational attainment, which serves as an economic resource that enables them to take control of their health and healthcare needs (35). Educated women are more aware of the health benefits of accessing maternity care during pregnancy, childbirth, and postpartum, as well as newborn and child healthcare (36). This result showed the importance of giving value to women's education by having early ANC visits. Education equips pregnant women with the knowledge of where, when, and how to access ANC services.

The region where women lived was a significant contributor to the inequality gap. Previous studies have focused on the impact of urban/rural residency on socioeconomic inequality for women. The literature did not assess regional disparities contributing to timely ANC visit, especially in equality aspect. Instead, the studies focused on residence in terms of urban vs. rural. However, this study showed the contribution of region to socioeconomic inequality in timely ANC visit. Women residing in small peripheral and metropolitan regions significantly contributed to the inequality in early ANC visits. Women in developing areas of Ethiopia encounter difficulties in obtaining healthcare services (37). In less developed areas of Ethiopia (small peripheral and metropolitan regions) where the majority of inhabitants are pastoralists without permanent residences, establishing health facilities and delivering services presents a challenge (38). This might be the reason for disparity for timely initiation of ANC visit. The other possible reason might be women in these regions had low economic status, scarcity of infrastructure to reach health service, long distance to health facility, low education status and other cultural factors result not starting ANC visit timely and inequality gaps. The distance to healthcare facilities is commonly cited as the primary obstacle preventing women from timely accessing and utilizing healthcare services (39).

### Limitation of the study

This study was a cross-sectional survey. The cross-sectional survey captures data at a single point in time and provide snapshot of the population at a particular moment. This cross-sectional study may not adequately represent population dynamics. Due to this limitation, the findings may not be generalized to the findings of other settings. However, this limitation can be outweighed by a larger sample size, and this study finding was generalizable to Ethiopian pregnant women due to the adequate sample size the inclusion of different regions and demographic groups of the country. Therefore, the limitation of this study is that temporal relationship cannot be determined.

The other limitation was the limited number of variables to be assessed for our study. The secondary nature of the data limited the choice of variables. All variables were not found in the EMDHS 2019 dataset. Important factors, including media exposure, the woman's and husband's occupations, the distance to healthcare access, and gravidity, were absent from the mini-version of Ethiopian demographic health survey data that influence the timing of ANC. These missing variables may lead to incomplete analysis and conclusions about the factors influencing the inequalities in the timing of ANC visit. It is essential to consider a diverse range of factors that influence the inequality in the timing of ANC visit.

## Conclusion

According to the study, more than half (63%) of the ANC visits were timely, which is a positive result. However, efforts should be made to achieve the SDG target. The wealth status, educational status, and region were significant factors to inequality in the timing of ANC visit. Working on socioeconomic inequalities is crucial to addressing the timing of ANC services at the recommended time. Improving women's wealth and education and narrowing the inequality gap contributed to improving the health status of women and their children. Any aspects of strategies and implementations targeted to early ANC visit should be focused on the determinants of socioeconomic inequalities.

### Recommendations

It is recommended to have policies that provide financial support, which can help minimize the impact of socioeconomic disparities on the timing of ANC visit. Providing comprehensive health education programs targeting women of reproductive age, focusing on the benefits of timely ANC visit and consequence of late visits, should be considered for achieving WHO-recommended timing of ANC visit. For regional disparities, outreach programs to deliver ANC services for women who live in small peripheral and metropolitan regions may tackle the gaps of inequalities. Depending on this study finding, we recommend researchers to consider qualitative study to explore specific barriers influencing the timing of ANC visits among different socioeconomic groups. In addition better for researchers to conduct geospatial analysis techniques to map the distribution of timing of ANC in relation to different socioeconomic areas. This helps to identify regional disparities and inform targeted resource allocation and infrastructure development to improve access to timely ANC services among marginalized regions of Ethiopia. Lastly, evidence based interventional studies are recommended to promote equitable access and timely initiation of ANC service.

## Data availability statement

The datasets presented in this study can be found in an online repository. The names of the repository and accession number(s) can be found below: https://www.dhsprogram.com.

## Ethics statement

Ethical approval was not required for the studies involving humans because not applicable because of secondary data. The studies were conducted in accordance with the local legislation and institutional requirements. Written informed consent for participation was not required from the participants or the participants' legal guardians/next of kin in accordance with the national legislation and institutional requirements because only verbal consent was obtained.

## Author contributions

AK: conceptualized the study and was involved in the design, analysis, interpretation, and manuscript writing. EF, SW, TAB, AZ, and TT: data analysis, manuscript editing. DA, SF, WN, TBB, BA, and AZ: manuscript writing, and editing. All authors contributed to the article and approved the submitted version.
